# Phospholipid Phosphatase 3 (*PLPP3*) Induces Oxidative Stress to Accelerate Ovarian Aging in Pigs

**DOI:** 10.3390/cells13171421

**Published:** 2024-08-25

**Authors:** Hongyan Quan, Yixuan Guo, Shuo Li, Yao Jiang, Qingpeng Shen, Yingting He, Xiaofeng Zhou, Xiaolong Yuan, Jiaqi Li

**Affiliations:** 1 Guangdong Laboratory of Lingnan Modern Agriculture, National Engineering Research Center for Breeding Swine Industry, State Key Laboratory of Swine and Poultry Breeding Industry, Guangdong Provincial Key Laboratory of Agro-Animal Genomics and Molecular Breeding, College of Animal Science, South China Agricultural University, Guangzhou 510642, China; quanhongyan2022@163.com (H.Q.);; 2 School of Medical, Molecular and Forensic Sciences, Murdoch University, Murdoch, WA 6150, Australia; 3 Shenzhen Branch, Guangdong Laboratory for Lingnan Modern Agriculture, Genome Analysis Laboratory of the Ministry of Agriculture, Agricultural Genomics Institute at Shenzhen, Chinese Academy of Agricultural Sciences, Shenzhen 518120, China; 4 Centre for Healthy Ageing, Health Futures Institute, Murdoch University, Murdoch, WA 6150, Australia

**Keywords:** ovarian aging, *PLPP3*, oxidative stress, ferroptosis, autophagy, pigs

## Abstract

Ovarian aging results in reproductive disorders and infertility in mammals. Previous studies have reported that the ferroptosis and autophagy caused by oxidative stress may lead to ovarian aging, but the mechanisms remain unclear. In this study, we compared the morphological characteristics between the aged and young ovaries of pigs and found that the aged ovaries were larger in size and showed more corpora lutea. TUNEL assay further showed that the apoptosis level of granulosa cells (GCs) was relatively higher in the aged ovaries than those in young ovaries, as well as the expressions of autophagy-associated genes, e.g., *p62*, *ATG7*, *ATG5*, and *BECN1*, but that the expressions of oxidative stress and aging-associated genes, e.g., *SOD1*, *SIRT1*, and *SIRT6*, were significantly lower. Furthermore, the RNA-seq, Western blotting, and immunofluorescence suggested that phospholipid phosphatase 3 (PLPP3) protein was significantly upregulated in the aged ovaries. *PLPP3* was likely to decrease the expressions of *SIRT1* and *SIRT6* to accelerate cellular senescence of porcine GCs, inhibit the expressions of *SOD1*, *CAT*, *FSP1*, *FTH1*, and *SLC7A11* to exacerbate oxidative stress and ferroptosis, and arouse autophagy to retard the follicular development. In addition, two SNPs of *PLPP3* promoter were significantly associated with the age at puberty. g.155798586 (T/T) and g.155798718 (C/C) notably facilitated the mRNA and protein level of *PLPP3.* In conclusion, *PLPP3* might aggravate the oxidative stress of GCs to accelerate ovarian aging, and two molecular markers of *PLPP3* were identified for ovarian aging in pigs. This work not only contributes to investigations on mechanisms for ovarian aging but also provides valuable molecular markers to postpone ovarian aging in populations.

## 1. Introduction

Ovarian aging is characterized by a reduction in follicular number and decay in oocyte quality, thus leading to a gradual decline in the fertility of females [1,2]. In mammals, the causes of ovarian aging are mainly attributed to the imbalance of antioxidant systems [3]. As the major driver of ovarian aging, oxidative stress declines the oocyte quality and brings mitochondrial dysfunction and cellular apoptosis in females [4]. Additionally, oxidative stress may initiate female reproductive pathology, which mainly causes follicular abnormally atresia, lower fertilization rate, and reproductive disease [5]. Excessive accumulation of reactive oxygen species (ROS) induced by oxidative stress results in apoptosis of granulosa cells (GCs) [6], boosts the degeneration of the corpus luteum and leads to follicular atresia in pigs [7]. Moreover, oxidative stress induces apoptosis and cellular senescence of GCs and contributes to infertility in women [8], as well as ovarian dysfunction in mice [9]. Nevertheless, the underlying mechanisms behind oxidative stress regulating ovarian aging remain unclear.

Ferroptosis is an iron (Fe) and ROS-dependent form of non-apoptotic cell death, and the excessive ROS caused by oxidative stress is an essential cause of ferroptosis [10]. Ferroptosis is characterized by the accumulation of lipid reactive oxygen species and iron, thus forming lipid hydroperoxides [11,12]. Ferroptosis of ovarian GCs aggravates ovarian dysfunction and contributes to polycystic ovary syndrome in women [13]. Additionally, as a major type of programmed cell death and type II cell death [14], autophagy digests the misfolded proteins in the lysosomes. It clears damaged organelles, such as mitochondria and peroxisomes [15]. It is highly recognized that autophagy maintains cellular and tissue homeostasis [16]. Autophagy protects diverse organisms against aging, and autophagic activity declines with natural aging [17]. It has been reported that autophagy retards aging in cardiovascular and heart of humans [18].

Nevertheless, excessive or dysfunctional autophagy is responsible for the death of porcine GCs induced by oxidative stress [19,20]. Interestingly, ROS-induced lipid peroxidation plays a vital role in regulatory cell death, including apoptosis, autophagy, and ferroptosis [21]. ROS leads to cardiolipin peroxidation to induce apoptosis in hepatocellular carcinoma [22], and ROS-induced lipid peroxidation activates mTORC1 signaling through the inhibition of AMPK and thus stimulates autophagy in cardiomyocytes [23]. Additionally, ROS induces autophagy to increase ferroptosis in fibroblastic cells [24]. Previous studies report that ferroptosis is one kind of autophagic cell death process, and autophagy induces ferroptosis by degradation of the iron storage protein ferritin [25] in fibroblasts and cancer cells [26]. These observations suggested that oxidative stress might induce ferroptosis and autophagy to regulate ovarian aging, but the mechanisms regarding these are undetermined.

Phospholipid phosphatase 3 (*PLPP3*) belongs to the phosphatidic acid phosphatase family and dephosphorylates the phospholipids into lipid signal products, which ROS typically oxidize into lipid hydroperoxides [27]. Previous studies have indicated lipid hydroperoxides induce cellular apoptosis, ferroptosis, and autophagy [21,28]. PLPP3 protein also plays a vital role in catalyzing the hydrolysis of lysophosphatidic acid, thus attenuating ovarian development in mice [29]. Therefore, we postulated that *PLPP3* might be involved in ovarian aging via regulating oxidative stress, ferroptosis, and autophagy. In this study, we aimed to explore the biological functions of *PLPP3* on oxidative stress, ferroptosis, and autophagy in GCs and investigate the role of *PLPP3* in follicular development. As the onset of puberty indicated the maturation of pig follicles, we further identified the associations between age at puberty and the potential molecular markers of *PLPP3*. These results might provide new targets for investigations on ovarian aging in mammals.

## 2. Materials and Methods

### 2.1. Aged and Young Ovaries Source

The aged and young ovaries of Landrace × Large White sows were used for this study, and these ovaries were sponsored by a pig farm (Guangzhou, China). The aged ovaries were removed from slaughtered sows (more than 6 parities), and the young ovaries were taken from gilts aged 5 months. The left ovaries of sows were stored in liquid nitrogen, while the right ovaries were stored in ice, and all of the ovaries were transported to our laboratory within 30 min. The ovaries stored in liquid nitrogen were used for RNA and protein extractions, and the ovaries stored in ice were placed in formaldehyde for subsequent experiments after being photographed. The experiments received approval from the Institutional Animal Care and Use Committee of South China Agricultural University (Approval number: SYXK 2022-0136).

### 2.2. Cell Culture and Treatments

The porcine primary GCs were cultured to explore the impact of *PLPP3* on the cellular functions of GCs. The isolation and culture of primary GCs were completed according to a previous study [30]. Briefly, ovaries of Landrace × Large White pigs (aged 5 months) were purchased from a local slaughterhouse (Guangzhou, China), soaked in phosphate buffered saline (PBS, Hyclone, Logan, UT, USA) containing 1% Penicillin Streptomycin (Invitrogen, Shanghai, China), and then transported to our laboratory in the ice box. The GCs were extracted from ovarian follicles (3–5 mm in diameter) with a 1 mL syringe. After being centrifuged at 820× *g* for 5 min and washed twice with PBS, the GCs were cultured in Dulbecco’s modified Eagle’s medium (DMEM, Hyclone) basal medium comprising 10% FBS (Hyclone) and incubated in a cell incubator (Thermo Scientific, Waltham, MA, USA) with a temperature of 37 °C alongside a 5% CO_2_ atmosphere. After GCs reaching 70–80% confluency, the overexpression plasmid (OE-*PLPP3*, 1000 ng/mL) and siRNA (si-*PLPP3*, 100 nM) of *PLPP3* were transfected into GCs using Lipofectamine™ 3000 (Thermo Scientific) for 24 h. Then, the RNA and protein were extracted from GCs for qRT-PCR and Western blotting analysis. These GCs were also used for subsequent cell phenotype and functional analysis. The *PLPP3* amplification primer is F: 5′-ATGCTGATGGTCCTCCTTGTATC-3′, R: 5′-CTACACCATGTTGTGGTGATTGTT-3′, and the product length is 831 bp. The siRNA sequence of *PLPP3* is 5′-CTGATGGTCCTCCTTGTAT-3′.

### 2.3. RNA-Seq Library Construction and Analysis

RNA-seq library construction was conducted by BGI Tech (Wuhan, China). TRIzol Reagent (Invitrogen, Carlsbad, CA, USA) was used to extract the total RNA from the aged and young ovaries of pigs and porcine GCs. The RNA samples were purified by Dynabeads^®^ mRNA Purification Kit (Invitrogen), and the Library Preparation VAHTS mRNA Capture Beads (Vazyme MGI, Nanjing, China) were applied to construct the cDNA library for RNA-Seq on the DNBSEQ platform (BGI) [31].

Reads were aligned to the reference genome (Sscrofa11.1/susScr11) gene set using HISAT2 [32], the transcriptome assembly was conducted using StringTie [33], and the DEGs were called by R package DESeq2 software (v.1.34.0) [34]. The transcripts with |log2FC| > 1 and *p* < 0.05 were considered DEGs. The volcano plots were used to visualize the hierarchical clustering of DEG patterns. Gene Ontology (GO) [35] was employed to analyze the GO enrichment, and a significance threshold of *p* < 0.05 was considered.

### 2.4. Genetic Analysis and Puberty Detection

The DNA extracted from 142 Duroc × Chinese Black Pigs was used for genetic analysis. The onset of sow’s puberty was identified according to the standing reflex under back-pressure test and contact from boars [36]. Pubertal signs were recorded twice per day by observing the vulva reaction and standing reflex from these 142 sows.

The genotype frequency and allele frequency of SNPs in the *PLPP3* promoter were calculated. The formula was as follows:

$$F_{AiAj}=\frac{AiAj}{n}\times 100\%$$

$$F_{Ai}=F_{AiAi}+\frac{1}{2}\sum F_{AiAj}$$

where $F_{AiAj}$ was genotype frequency, AiAj was the number of individuals, *n* was the total sample number of the population, and $F_{Ai}$ was the allele frequency.

The genetic association analysis between *PLPP3* SNPs and the age at puberty of 142 Duroc × rural black pigs (Zhanjiang, China) was conducted using R language (v4.03) and based on the general linear model (GLM). The model was as follows:

$$Y_{ij}=L+G_{j}$$

where $Y_{ij}$ was the age at puberty observed by the *i*-th animal, and *L* was the average age at puberty; $G_{j}$ represented genotype effects. The statistical data were expressed as “mean ± standard deviation”.

### 2.5. Luciferase Reporter Assays

KGN cells were seeded into 6-well plates and cultured for 24 h. The luciferase reporter constructs containing *PLPP3* promoter with SNP g.155798586 (T/T), g.155798586 (C/C), g.155798718 (C/C), and g.155798718 (T/T) sites were sub-cloned into the pGL3 vectors by Genecreate Co., Ltd. (Wuhan, China). The plasmids containing promoter-reporter and pRL-TK Renilla control plasmid were co-transfected into KGN cells in a 39:1 ratio using Lipofectamine™ 3000 (Thermo Scientific). After 24 h culture, the cells were lysed and harvested, and then the luciferase activity was measured by utilizing the Dual Luciferase Assay System (Beyotime, Shanghai, China). The data were obtained by Synergy 2 multifunctional microplate reader (BioTek, Winooski, VT, USA), and the luciferase activity was normalized to the Renilla luciferase activity.

### 2.6. Real-Time Quantitative PCR (qRT-PCR)

The total RNA was extracted using TRIzol reagent (Invitrogen). cDNA was generated with Hifair^®^ Advance Fast 1st Strand cDNA Synthesis Kit (Yisheng, Shanghai, China). Quantitative real-time PCR was conducted using Hieff^®^ qPCR SYBR Green Master Mix (Yisheng) on a CFX96 Touch Real-Time PCR system (Bio-Rad, Berkeley, CA, USA). Relative levels of mRNA were determined by the 2^−ΔΔct^ method with normalization to glyceraldehyde phosphate dehydrogenase (*GAPDH*). The primer sequences applied are listed in Supplementary Table S1.

### 2.7. Immunofluorescence, Hematoxylin–Eosin Staining (HE), and TUNEL Assay

Immunofluorescence: The fresh ovaries were fixed in 4% paraformaldehyde for 48 h and then rinsed with running water 3 times, each time for 5 min. The ovaries were embedded in melted paraffin at 55 °C after being dehydrated with gradient ethanol (70, 80, 90, 95, and 100%) and treated with 100% xylene, respectively. The embedded ovaries were then cut into maximum transverse sections, and each section was 3 μm in thickness. Paraffin-embedded sections were blocked with 3–5% bovine serum albumin (BSA, Thermo Scientific) for 30 min, and then stained with the corresponding primary antibodies at 4 °C overnight and incubated with fluorescent-labeled secondary antibodies at 37 °C for 3 h. Finally, the images of sections were captured by Nikon ECLIPSE Ti2 fluorescence microscope (Nikon, Tokyo, Japan), and the fluorescence intensity was measured using ImageJ software (V1.8.0.345, National Institutes of Health, Stapleton, NY, USA). The ratio of gray value of green (red) fluorescence to the area of blue fluorescence quantified the mean fluorescence intensity. All of the antibodies are listed in Supplementary Table S2.

HE staining: The ovaries were embedded in paraffin and cut into maximum transverse sections, and each section was 3 μm thick. Then, the paraffin sections were stained with hematoxylin for 1 min, followed by eosin for a few seconds. After being washed with running water, the sections were observed by a Nikon ECLIPSE Ti2 fluorescence microscope (Nikon).

TUNEL assay: A TUNEL Apoptosis Assay Kit (Beyotime) was used to measure TUNEL-positive sections in porcine ovaries. Briefly, the ovarian paraffin sections were incubated with xylene for 9 min, followed by ethanol for 7 min, and then protease K for 14 min. After being washed with PBS, the sections were incubated with terminal deoxynucleotidyl transferase enzyme and Cy3-labeled dUTP mixture provided by the Assay Kit for 1.5 h at 37 °C. The fluorescence images of ovaries were then captured using Nikon ECLIPSE Ti2 fluorescence microscope (Nikon), and the apoptotic cells were stained green. The fluorescence intensity was quantified by ImageJ software (V1.8.0.345, National Institutes of Health), and the apoptosis level was quantified by the mean fluorescence intensity (the ratio of gray value of green fluorescence to the area of blue fluorescence).

### 2.8. ROS Detection

The intracellular ROS level was detected using Reactive Oxygen Species Assay Kit (Beyotime). DCFH was employed as a probe to detect the generation of intracellular ROS, and the hydrolyzation of DCFH-DA obtained DCFH. GCs were planted in a 96-well plate (Thermo Scientific) for 24 h and transfected with overexpression plasmid and siRNA (Synthesized by Dongze Biotechnology, Guangzhou, China) of *PLPP3* for another 24 h. After removing the cell culture medium, 100 μL 10 μM DCFH-DA label was added per well, and GCs were then incubated at 37 °C for 40 min in dark. The GCs in positive control were treated with the Rosup reagent, while the GCs in negative control were not treated. Afterwards, the GCs were washed twice with PBS to remove the ROS label. The fluorescence images were collected by a Nikon ECLIPSE Ti2 fluorescence microscope (Nikon). The ROS level was quantified by the optical density (OD) at 495 nm on a Synergy 2 multifunctional microplate reader (BioTek, USA).

### 2.9. Autophagy Level Detection

Tandem fluorescent mRFP-GFP-LC3 (Ad-GFP-LC3B) (Hanbio, Shanghai, China) was employed to measure the autophagy level in GCs, which was quantified by the number of autophagosomes and autolysosomes according to the fluorescent LC3 puncta. GCs were cultured in 48-well plates, and when the cells reached 50% confluence, Ad-GFP-LC3B was transfected into GCs. Then, 4 h later, cells were transfected with the overexpression plasmid and siRNA of *PLPP3* for another 24 h. After being rinsed with PBS, the intracellular fluorescent LC3 puncta were observed with a laser-scanning confocal microscope (TCS SP8, Leica, Heidelberg, Germany). Experiments were performed in triplicate, and three views were chosen randomly per well for photography at a magnification of 80×.

### 2.10. SA β-Gal Assay

Senescence β-Galactosidase Staining Kit (Beyotime) was employed to evaluate the SA β-gal activity in GCs. The activity of SA β-gal is increased during cellular aging. Using X-Gal as a substrate, a deep blue product is generated under SA β-gal enzyme catalysis. Briefly, cells were fixed with SA β-gal fixation fluid at RT for 12–15 min and then washed with PBS 3 times. After being stained with X-gal solution overnight at 37 °C, cell images were captured by Nikon ECLIPSE Ti2 fluorescence microscope (Nikon), and the number of SA β-gal-positive cells (deep blue cells) was counted by ImageJ software (V1.8.0.345, National Institutes of Health).

### 2.11. Malondialdehyde (MDA) Detection

Malondialdehyde (MDA) Colorimetric Assay Kit (Cell Samples) (Elabscience, Wuhan, China) was applied to measure the level of MDA. GCs were transfected with overexpression plasmid and siRNA of *PLPP3* for 24 h. The harvested cells were lysed with Extracting Solution provided by the Assay Kit, and then, a water bath was performed at 100 °C for 40 min. After being cooled to RT, the samples were centrifuged at 1078× *g* for 10 min. The absorbance of the samples was measured at 532 nm using Synergy 2 multifunctional microplate reader (BioTek).

### 2.12. Fe Detection

The Cell Total Iron Colorimetric Assay Kit (Elabscience) detected the Fe level. The harvested cells were lysed on ice with Buffer Solution provided by the Assay Kit for 10 min. After being centrifuged at 15,000× *g* for another 10 min, the supernatant was collected and incubated with Chromogenic Solution provided by the Kit at 40 °C for 35 min. The samples’ optical density (OD) was measured by Synergy 2 multifunctional microplate reader (BioTek) at 593 nm.

### 2.13. Determination of Mitochondrial Membrane Potential

A mitochondrial membrane potential assay kit with JC−1 (Beyotime) was utilized to measure the mitochondrial membrane potential changes in GCs. GCs were incubated with JC−1 working solution at 37 °C for 20 min. After being washed with JC−1 staining buffer 2 times, GCs were observed under a Nikon ECLIPSE Ti2 fluorescence microscope (Nikon). With mitochondrial membrane potential changes, JC−1 exists in two forms: the cytoplasmic JC−1 monomers and mitochondrial JC−1 aggregates. When the mitochondrial membrane potential increases, mitochondrial JC−1 aggregates emit red fluorescence. However, when the mitochondrial membrane potential decreases, the released JC−1 from mitochondria forms JC−1 monomers in the cytoplasm, producing green fluorescence. Therefore, the red-to-green fluorescence intensity ratio measured the relative mitochondrial membrane potential.

### 2.14. Western Blot Assay (WB) and Antibodies

RIPA Lysis Buffer (Thermo Scientific) containing 2% protease inhibitor (BioSharp, Sichuan, China) was utilized to extract the total proteins, and BCA Protein Assay Kit (BioVision, Milpitas, CA, USA) was employed to measure the concentrations of proteins. Equal amounts of proteins (20 μg) were added per lane. SDS-PAGE gels separated proteins with different sizes at 140 V for 45 min. Then, the SDS-PAGE gels that contained proteins were electrotransferred to the polyvinylidene fluoride (PVDF) membranes (Bio-Rad, USA) by utilizing eBlot™ L1 membrane converter (GenScript, Nanjing, China). The PVDF membranes were then sealed with 5% skimmed milk powder (diluted with Tris-buffered saline-Tween (TBST)) for 2 h at RT. After being washed with TBST, the membranes were incubated with primary antibodies overnight at 4 °C. After being washed with TBST for 9 min (×3 times), the membranes were then incubated with secondary antibodies at RT for 1.5–2 h. The protein bands were visualized by Tanon 4600 SF (Tanon, Shanghai, China), and ImageJ software (V1.8.0.345, National Institutes of Health) was used to measure the protein band intensity. All of the antibodies are listed in Supplementary Table S3.

### 2.15. Statistical Analysis and Reproducibility

The number of biological independent samples and replicates of experiments are included in the figure legends. For RNA-seq, three independent biological replicates were included. All the data met the statistical test assumptions, including whether normality and equal variances were formally tested. For normally distributed data, data were reported as mean ± SD. Statistical significance was evaluated by a two-tailed *t*-test depending on data normality using the GraphPad Prism software (GraphPad Software, v. 8.0.2.263, La Jolla, CA, USA), and statistical significance is indicated in the figures.

## 3. Results

### 3.1. Morphological Characteristics of the Aged and Young Ovaries

**Figure 1:**
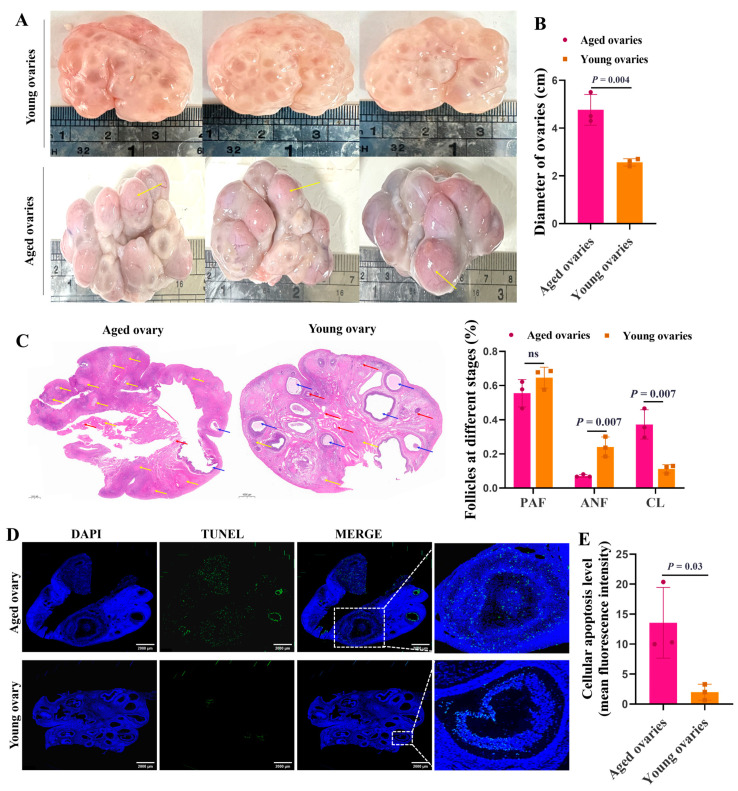
Morphological characteristics of the aged and young ovaries. Representative morphology images of the aged and young ovaries of sows (**A**) and the statistics of ovarian diameter (**B**). (**C**) Representative images of HE staining showing the ovarian morphology of sows (Scale bar: 1000 μm). The red arrows point to the preantral follicles (PAFs). The blue arrows point to antral follicles (ANFs). The yellow arrows point to the corpus luteum (CL). (**D**) TUNEL fluorescence assay of follicular GCs. The nuclei of TUNEL-positive (apoptotic) cells were stained green (Scale bar: 2000 μm). The blue indicates the TUNEL-negative signal. (**E**) The apoptosis level was quantified by the mean fluorescence intensity (the ratio of gray value of green fluorescence/the area of blue fluorescence). The graphs are shown as mean ± SD. The number of independent biological samples (represented as dots) used and the *p*-values are indicated in the graphs.

The aged ovaries were taken from sows of more than six parities, and the young ovaries were taken from gilts aged 5 months. The sizes of aged ovaries (4 to 6 cm in diameter) were larger than that of the young ones (2 to 3 cm in diameter) (Figure 1A,B). Compared to the young ovaries, the aged ovaries had abundant corpora lutea (yellow arrows) (Figure 1A). HE staining further supported that, compared to the aged ovaries, the young ovaries had more primary (red arrows) and antral follicles (blue arrows), and the rate of antral follicles was significantly increased (Figure 1C). Importantly, TUNEL assay showed that the aged ovaries exhibited higher apoptosis levels of GCs in antral follicles than the young ones (Figure 1D,E).

### 3.2. PLPP3 May Be Involved in Regulating Ovarian Aging via Oxidative Stress and Autophagy

**Figure 2:**
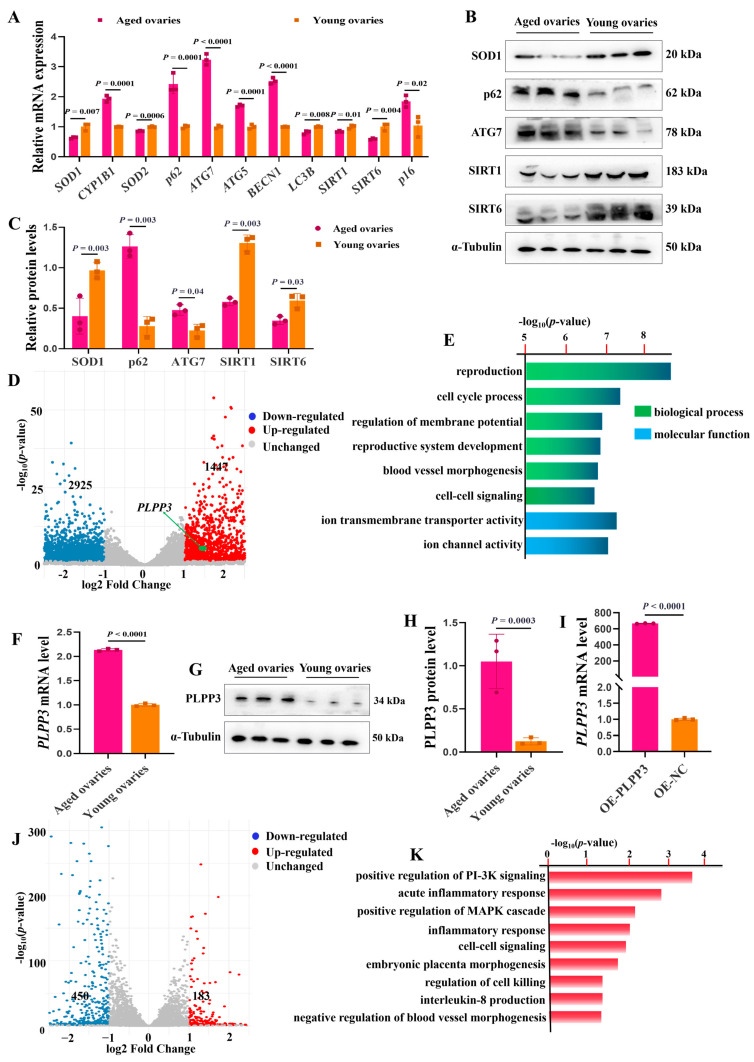
*PLPP3* may be involved in regulating ovarian aging. (**A**) The mRNA levels of oxidative stress-associated genes *SOD1*, *CYP1B1*, and *SOD2*, autophagy-associated genes *p62*, *ATG7*, *BECN1*, and *LC3B*, and aging-associated genes *SIRT1*, *SIRT6*, and *p16.* The protein levels of SOD1, p62, ATG7, SIRT1, and SIRT6 measured by Western blotting (**B**) and quantified by grayscale analysis (**C**). (**D**) The volcano plot showing the differentially expressed genes between the aged and young ovaries of sows (log2FC > 1, *p* ≤ 0.05). (**E**) Functional annotation of differentially expressed genes by GO analysis. (**F**) The mRNA level of *PLPP3* detected by qRT−PCR in sows’ aged and young ovaries. The protein level of PLPP3 measured by Western blotting (**G**) and quantified by grayscale analysis (**H**). (**I**) The mRNA level of *PLPP3* in GCs with *PLPP3* overexpression (OE−*PLPP3*, 1000 ng/mL). (**J**) The volcano plot showing the differentially expressed genes with *PLPP3* overexpression in GCs (log2FC > 1, *p* ≤ 0.05). (**K**) The functional annotation of differentially expressed genes by GO analysis. The graphs are shown as mean ± SD. The number of independent biological samples (represented as dots) used, and the *p*−values are indicated in the graphs.

The mRNA levels of Superoxide Dismutase 1, 2 (*SOD1*, *SOD2*), microtubule-associated protein 1 light chain 3 beta (LC3B), aging-associated markers sirtuin 1 (*SIRT1*), and sirtuin 6 (*SIRT6*) were remarkably downregulated in the aged ovaries of sows (Figure 2A). Meanwhile, the mRNA levels of cytochrome P450 family 1 subfamily B member 1 (*CYP1B1*), autophagy-related 7 (*ATG7*), autophagy-related 5 (*ATG5*), beclin 1 (*BECN1*), SQSTM1 sequestosome 1 (*p62*), and cyclin-dependent kinase inhibitor 2A (*p16*) were significantly upregulated (Figure 2A). In addition, the protein levels of SOD1, p62, SIRT1, and SIRT6 were remarkably decreased in the aged ovaries, but p62 and ATG7 were increased compared to those in the young ones (Figure 2B,C). These results suggested that oxidative stress and autophagy might regulate ovarian aging.

Subsequently, RNA-sequencing (RNA-seq) analysis revealed that 1447 genes were significantly upregulated, and 2925 genes were downregulated in the aged ovaries, compared to the young ones (Figure 2D). Gene Ontology (GO) analysis further exhibited that the differentially expressed genes were mainly enriched in the regulation of the reproductive process and membrane potential, and previous studies have suggested that the decreased membrane potential brought by mitochondrial dysfunction contributes to cellular senescence [37] (Figure 2E). Interestingly, RNA-seq analysis showed that *PLPP3* was significantly upregulated in the aged ovaries compared to the young ones (Figure 2D). Furthermore, in contrast to the young ovaries, the mRNA (Figure 2F) and protein levels (Figure 2G,H) of *PLPP3* were remarkably increased in the aged ovaries. *PLPP3* overexpression (Figure 2I) upregulated 183 genes and downregulated 450 genes in GCs compared to the control group (Figure 2J). These differentially expressed genes were mainly enriched in regulating inflammatory response, blood vessel morphogenesis, MAPK, and PI-3K signaling (Figure 2K), essential in regulating follicular development and ovarian aging [38,39].

### 3.3. PLPP3 Increases the Cellular Senescence of GCs

**Figure 3:**
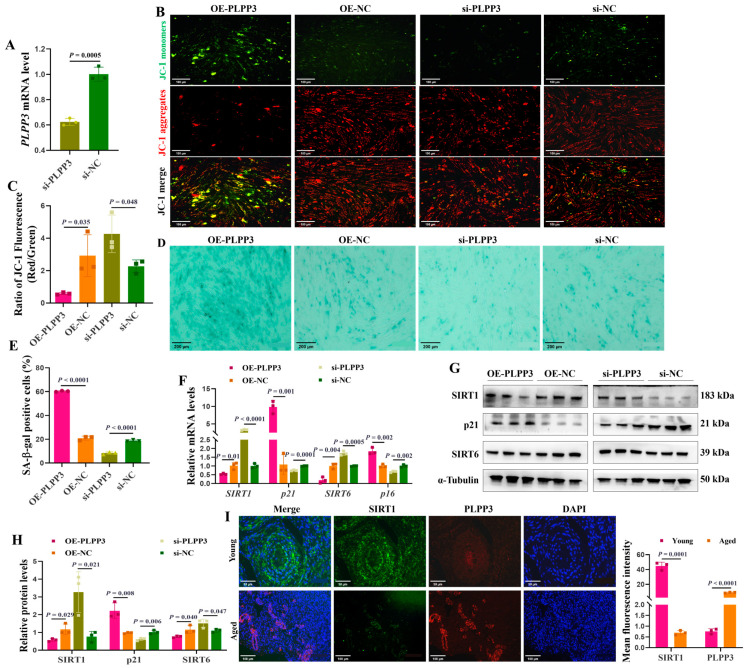
*PLPP3* may accelerate the senescence in GCs. (**A**) The mRNA level of *PLPP3* detected in porcine primary GCs with *PLPP3* knockdown (si-*PLPP3*, 100 nM). (**B**) Representative images of JC−1 staining showing the mitochondrial membrane potential in GCs. The stronger red fluorescence (JC−1 aggregates) represents higher mitochondrial membrane potential. When the mitochondrial membrane potential decreases, green fluorescence (JC−1 monomers) is generated (Scale bar: 100 μm). (**C**) The ratio of red and green fluorescence intensity quantified the mitochondrial membrane potential. Representative images of SA-β-Gal staining (Scale bar: 200 μm) (**D**), and the SA-β-Gal activity was quantified by the analysis of SA-β-Gal-positive cells (blue dots) in GCs (**E**). (**F**) The mRNA levels of *SIRT1*, *SIRT6*, *p16*, and *p21* detected by qRT-PCR. The protein levels of SIRT1, SIRT6, and p21 detected by Western blotting (**G**) and quantified by the gray value of protein bands (**H**). (**I**) The sub-cellular location and protein expressions of SIRT1 (green) and PLPP3 (red) assessed by immunofluorescence analysis in ovaries of sows (Scale bar: Young, 50 μm; Aged, 100 μm). The graphs are shown as mean ± SD. The number of independent biological samples (represented as dots) used and the *p*-values are indicated in the graphs.

*PLPP3* overexpression (Figure 2I) and knockdown (Figure 3A) were used to explore their impacts on cellular senescence in porcine primary GCs. *PLPP3* overexpression significantly decreased the mitochondrial membrane potential in GCs, as JC−1 aggregates yielded significantly weaker red fluorescence, while the JC−1 monomers yielded relatively stronger green fluorescence than the control (Figure 3B,C). Additionally, *PLPP3* overexpression increased the SA-β-Gal activity in GCs, while GCs with *PLPP3* knockdown effectively inhibited SA-β-Gal activity (Figure 3D,E). Furthermore, the mRNA (Figure 3F) and protein (Figure 3G,H) levels of senescence-associated markers *SIRT1* and *SIRT6* were found to be significantly suppressed in GCs with *PLPP3* overexpression. At the same time, the expression of cyclin-dependent kinase inhibitor 1A (*p21*) was increased. However, *PLPP3* knockdown remarkably prompted the expressions of *SIRT1* and *SIRT6* and inhibited *p21*. The immunofluorescence results showed that PLPP3 protein was upregulated in the aged ovaries compared to the young ovaries, while SIRT1 was downregulated (Figure 3I). Overall, these results indicated that *PLPP3* might increase cellular senescence in GCs.

### 3.4. PLPP3 Aggravates Oxidative Stress and Ferroptosis of GCs

**Figure 4:**
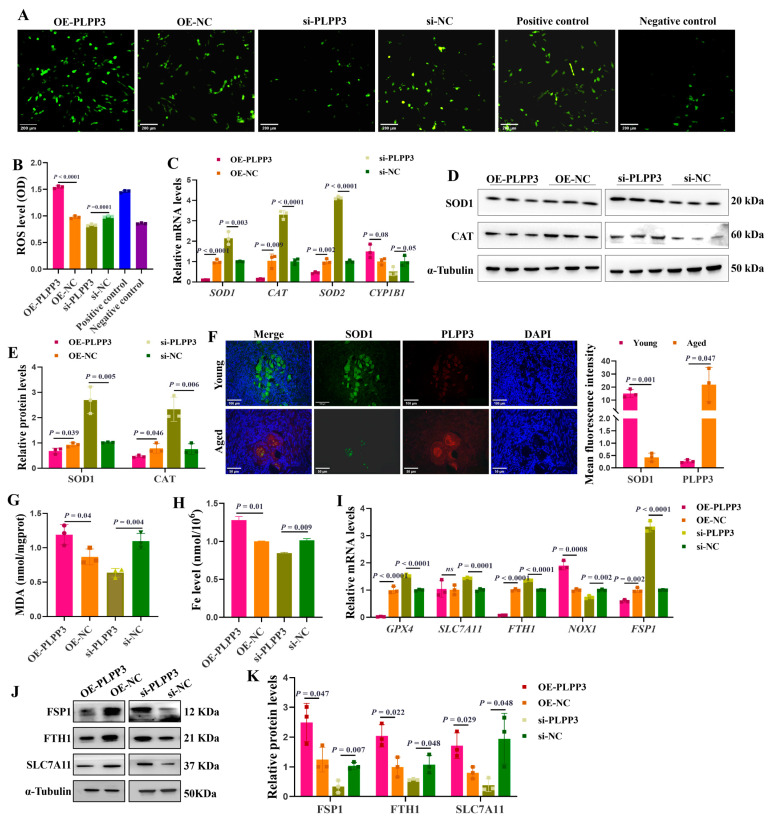
*PLPP3* aggravates the oxidative stress and ferroptosis in GCs. (**A**) Representative images of ROS level in GCs with *PLPP3* overexpression and *PLPP3* knockdown (Scale bar: 200 μm). (**B**) The ROS level was quantified with OD (optical density) at 495 nm. (**C**) The mRNA levels of *SOD1*, *CAT*, *SOD2*, and *CYP1B1* detected by qRT-PCR. The protein levels of SOD1 and CAT detected by Western blotting (**D**) and quantified by the gray value of protein bands (**E**). (**F**) The sub-cellular location and protein expressions of SOD1 (green) and PLPP3 (red) assessed by immunofluorescence analysis in ovaries of sows (Scale bar: Young, 100 μm; Aged, 50 μm). The measurement of MDA (**G**) and Fe (**H**) levels in GCs with *PLPP3* overexpression and *PLPP3* knockdown. (**I**) The mRNA levels of *GPX4*, *SLC7A11*, *FTH1*, *NOX1*, and *FSP1* detected by qRT-PCR. The protein levels of FSP1, FTH1, and SLC7A11 detected by Western blotting (**J**) and quantified by the gray value of protein bands (**K**). The graphs are shown as mean ± SD. The number of independent biological samples (represented as dots) used, and the *p*-values are indicated in the graphs.

As shown in Figure 4A,B, *PLPP3* overexpression significantly increased the accumulation of intracellular ROS in GCs. However, in the presence of *PLPP3* knockdown, the ROS level was notably decreased. The mRNA levels of *SOD1*, *CAT*, and *SOD2* were significantly reduced in GCs with *PLPP3* overexpression, accompanied by an increase in *CYP1B1* (Figure 4C). Besides, *PLPP3* overexpression remarkably downregulated the protein levels of SOD1 and CAT, while an upregulation of SOD1 and CAT was observed in GCs with *PLPP3* knockdown (Figure 4D,E). Correspondingly, the immunofluorescence results further revealed that PLPP3 was upregulated in the aged ovaries of sows but accompanied by a downregulation of SOD1 (Figure 4F).

Malondialdehyde (MDA) is a marker of oxidative stress-induced lipid peroxidation [40], and a significantly increased level of MDA was observed in GCs with *PLPP3* overexpression (Figure 4G). Additionally, *PLPP3* overexpression increased the level of Fe (Figure 4H), and the overload of Fe may promote lipid peroxidation and the accumulation of ROS and decrease the mitochondrial membrane potential [41]. However, *PLPP3* knockdown significantly decreased both the MDA and Fe levels in GCs (Figure 4G,H). The mRNA levels of ferroptosis suppressor genes glutathione peroxidase 4 (*GPX4*) [42], ferritin heavy chain 1 (*FTH1*) [43], and ferroptosis suppressor protein 1 (*FSP1*) [44] were remarkably downregulated in GCs with *PLPP3* overexpression, but the mRNA level of NADPH oxidase 1 (*NOX1*) was upregulated (Figure 4I). In addition, *PLPP3* overexpression significantly decreased the protein levels of ferroptosis suppressor proteins FSP1, FTH1, and solute carrier family 7 member 11 (SLC7A11) [45], while *PLPP3* knockdown obviously increased the expressions of FSP1, FTH1, and SLC7A11 (Figure 4J,K). Hence, these results suggested that *PLPP3* might arouse oxidative stress and ferroptosis to accelerate senescence in GCs.

### 3.5. PLPP3 Elevates the Autophagy Level in GCs

**Figure 5:**
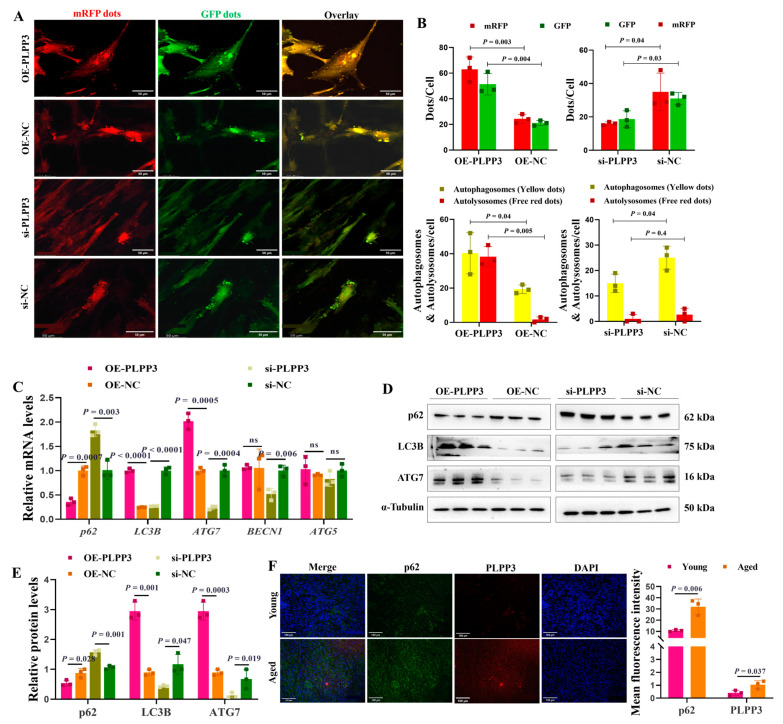
*PLPP3* increases the autophagy level in GCs. (**A**) Representative images of GFP-LC3 puncta in GCs with *PLPP3* overexpression and knockdown (Scale bar: 50 μm). (**B**) The autophagy level was quantified by the number of autophagosomes (yellow dots) and autolysosomes (free red dots). (**C**) The mRNA levels of *p62*, *LC3B*, *ATG7*, *BECN1*, and *ATG5* detected by qRT-PCR. The protein levels of p62, LC3B, and ATG7 detected by Western blotting (ns = not significant) (**D**) and quantified by the gray value of protein bands (**E**). (**F**) The sub-cellular location and protein expressions of p62 (green) and PLPP3 (red) assessed by immunofluorescence analysis in ovaries of sows (Scale bar: 100 μm). The graphs are shown as mean ± SD. The number of independent biological samples (represented as dots) used, and the *p*-values are indicated in the graphs.

The tandem mRFP-GFP-LC3 adenovirus was employed to measure the impact of *PLPP3* on autophagy in GCs. We identified that *PLPP3* overexpression remarkably increased the number of autophagosomes and autolysosomes, thereby enhancing the autophagy flux in GCs (Figure 5A,B). However, *PLPP3* knockdown significantly attenuated the autophagy flux by reducing the number of autophagosomes and autolysosomes (Figure 5A,B). Correspondingly, *PLPP3* overexpression obviously increased the mRNA levels of *LC3B*, *ATG7*, *BECN1*, and *ATG5*, while decreasing the mRNA level of *p62* (Figure 5C). Additionally, the protein levels of LC3B and ATG7 were notably increased by *PLPP3* overexpression, while the protein level of p62 was remarkably decreased (Figure 5D,E). As expected, *PLPP3* knockdown obviously suppressed the expressions of LC3B and ATG7 and prompted the expression of p62 (Figure 5D,E), suggesting that the overexpression of *PLPP3* might arouse a protective autophagy response against oxidative stress damage in GCs. The immunofluorescence results further demonstrated that PLPP3 was upregulated in the aged ovaries and accompanied by an upregulation of p62 (Figure 5F), which indicated a low autophagy level in the aged ovaries. These findings suggested that *PLPP3* might trigger oxidative stress-induced autophagy, thus contributing to the ovarian aging of sows.

### 3.6. g.155798586T>C and g.155798718C>T May Be Molecular Markers for Follicular Development

**Figure 6:**
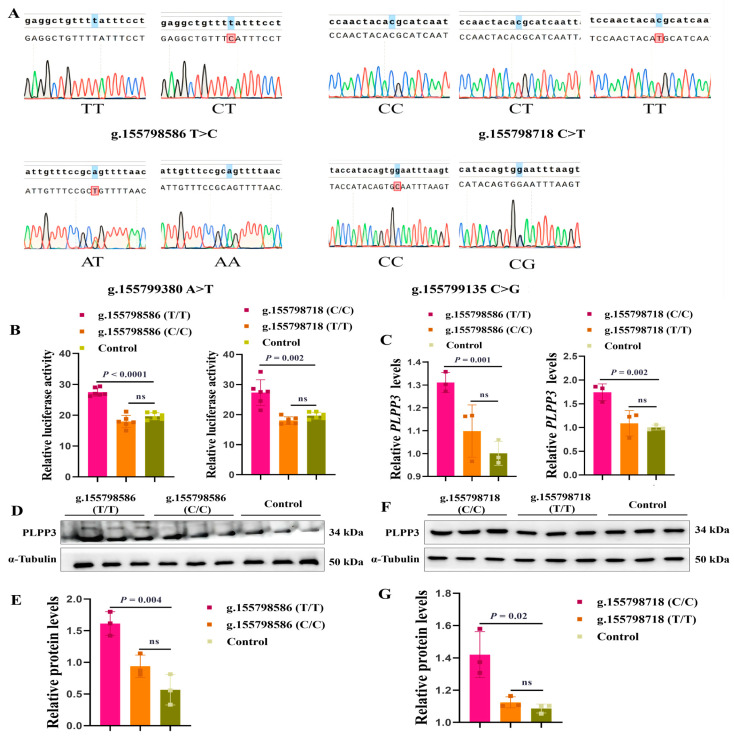
g.155798586 T>C and g.155798718 C>T might be molecular markers for follicular development. (**A**) The genotypes of four SNPs in the promoter region of *PLPP3.* The first line is the reference sequence, and the second line is the aligned sequence. The marked bases are inconsistent with the reference sequence. (**B**) The dual luciferase activities of g.155798586 (T/T), g.155798586 (C/C), g.155798718 (C/C), and g.155798718 (T/T). (**C**) The mRNA level of *PLPP3* detected by qRT-PCR (ns = not significant). Western blotting showing protein levels of PLPP3 in GCs transfected with g.155798586 (T/T) and g.155798586 (C/C) (**D**,**E**), g.155798718 (C/C) and g.155798718 (T/T) plasmids (**F**,**G**) (ns = not significant). The graphs are shown as mean ± SD. The number of independent biological samples (represented as dots) used and the *p*-values are indicated in the graphs.

As the onset of puberty is a hallmark of the maturation of follicles [46,47], we further explored the genetic association analysis between the SNPs of the *PLPP3* promoter and age at puberty. Our genetic polymorphism analysis (n = 142) revealed four SNPs (g.155798586 T>C, g.155798718 C>T, g.155799380 A>T, and g.155799135 C>G) in the promoter of *PLPP3* (Figure 6A). The genotype frequency and allele frequency are listed in Table 1. Interestingly, g.155798586 T>C and g.155798718 C>T were significantly correlated with the puberty of sows (*p* < 0.01) (Table 2). The phenotype of TT (135.2 ± 10.994 d) was significantly delayed compared to that of CT (126.7 ± 7.499 d) at g.155798586, and the phenotype of CC (138.7 ± 9.519 d) was significantly delayed compared to that of CT (129.0 ± 10.058 d) and TT (118.0 ± NA d) at g.155798718 (Table 2).

Subsequently, we cloned two genotypes of g.155798586 and g.155798718 (C and T) into pGL3 reporter plasmids, and the luciferase activity of these plasmids was detected. Results showed that the luciferase activity of g.155798586 (T/T) was significantly higher than that of g.155798586 (C/C), and the luciferase activity of g.155798718 (C/C) was significantly higher than that of g.155798718 (T/T) (Figure 6B). We further found that the mRNA (Figure 6C) and protein levels of *PLPP3* were remarkably increased by g.155798586 (T/T) (Figure 6D,E) and g.155798718 (C/C) (Figure 6F,G), while g.155798586 (C/C) and g.155798718 (T/T) showed insignificant effects on the mRNA and protein expressions of *PLPP3*. These results suggested that g.155798586T>C and g.155798718C>T might regulate the expression of *PLPP3* and follicular development.

## 4. Discussion

Ovarian aging is the basis of reproductive aging, which refers to the gradual decline in the quantity and quality of follicles and oocytes [48], resulting in premature ovarian failure [49] and infertility in humans [2]. Oxidative stress is critical for rats’ follicular growth and corpus luteum formation [50]. Still, its elevation is the primary cause leading to follicular failure and ovarian aging in mice [51]. Here, we found that the aged ovaries had relatively more corpus luteum than the young ones, while the young ovaries had abundant primary follicles and antral follicles (Figure 1A,B). In the aged ovaries, we also observed a decrease in antioxidant SOD1 (Figure 2A), which is responsible for defending oxidative stress induced by ROS [52]. The imbalance between antioxidants and ROS leads to cellular apoptosis and declined ovarian oocyte quality, ultimately promoting women’s ovarian aging [4]. Our findings suggested that oxidative stress might be essential in affecting ovarian aging.

Subsequently, we found that *PLPP3* was significantly upregulated in the aged ovaries compared to that in the young ovaries (Figure 2D,F,G). RNA-seq further revealed potential pathways through which *PLPP3* influenced the regulation of follicular development, including inflammation, MAPK signaling, and regulation of blood vessel morphogenesis (Figure 2K). The genes related to inflammatory responses have been significantly enriched in aged mice [53]. *PLPP3* might be a potential ovarian aging regulator via oxidative stress induction. Studies have shown that *PLPP3* catalyzes the dephosphorylation of phospholipids into lipid signal products, which are typically oxidized by ROS into lipid hydroperoxides [27], thereby inhibiting the follicular development of mice [54], bovine, and humans [55,56]. Meanwhile, *PLPP3* impairs follicular development of mice by dephosphorylating lipid substrate’s LPA [57]. More importantly, our genetic polymorphism analysis identified that the g.155798586 T>C and g.155798718 C>T in the *PLPP3* promoter might serve as candidate molecular markers for follicular development, showing a significant correlation with age at puberty (Table 2). The g.155798586 (T/T) and g.155798718 (C/C) notably boosted the expression of *PLPP3* (Figure 6C–G). The ALGGEN PROMO tool predicted that 13 transcription factors might bind to the g.155798586 T>C locus, while 8 transcription factors were identified to bind to g.155798718 C>T locus. It is hypothesized that these transcription factors may be involved in regulating *PLPP3*.

Here, we demonstrated that overexpression of *PLPP3* significantly increased cellular senescence in GCs (Figure 3), along with elevated ROS level (Figure 4A,B) and decreased expressions of antioxidants SOD1 and CAT (Figure 4C–E). SOD enzymes play a crucial role in removing ROS and are commonly found in mammalian ovaries [58]. Knocking out *SOD1* inhibits follicular development, resulting in an increase in primary follicles and a decrease in mature follicles in women [59]. Additionally, *PLPP3* overexpression induced lipid hydroperoxides and ferroptosis by elevating the accumulation of MDA and Fe in GCs (Figure 4G,H). Since excessive ROS, due to oxidative stress, is a major contributor to ferroptosis, it is suggested that *PLPP3* may accelerate the senescence of GCs by disrupting the balance between antioxidants and ROS to aggravate oxidative stress.

The accumulation of autophagosomes leads to cell death in follicular GCs of rats [60], while oxidative stress induces autophagy, apoptosis, and inflammation responses in mammals [61,62]. Moderate autophagy is beneficial for cell survival by removing oxidized products and damaged mitochondria caused by oxidative stress, thereby reducing the excessive accumulation of ROS [63]. Interestingly, excessive autophagy is responsible for the death of GCs induced by oxidative stress [19]. In addition, autophagy participates in regulating ovarian function, as knockout of the autophagy induction gene *Atg7* in mouse germ cells leads to subfertility in female mice [64]. The autophagy substrate, p62, negatively regulates autophagy by activating mTOR [65], but its absence leads to mTOR complex inactivation and exacerbation of autophagy in humans [66]. Here, we revealed that *PLPP3* increased the autophagy level in GCs (Figure 5A,B), indicating that *PLPP3* exacerbated oxidative stress to induce autophagy and ovarian aging. Nevertheless, we should note that the use of FCs for cultivating GCs may have an impact on cell proliferation, as well as on the production of oestradiol and progesterone, and even on cellular luteinization in porcine GCs [67].

**Figure 7:**
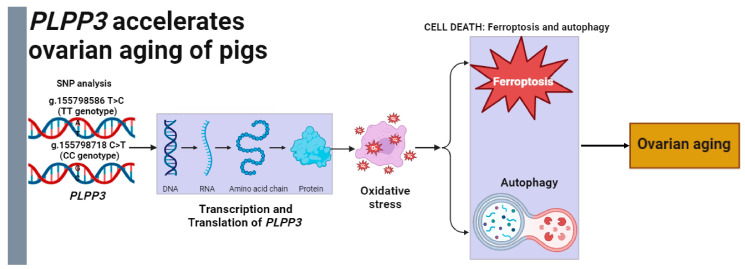
Model for *PLPP3* regulating oxidative stress to accelerate ovarian aging of pigs. The g.155798586 T>C and g.155798718 C>T might be the practical molecular markers for ovarian aging and *PLPP3*-induced oxidative stress, ferroptosis, and autophagy to accelerate ovarian aging of sows.

In conclusion, our study indicated that *PLPP3* might be a potential regulator of ovarian aging (Figure 7). Specifically, *PLPP3* effectively aggravated oxidative stress, ferroptosis, and autophagy in GCs, leading to an acceleration of cellular senescence. Furthermore, the g.155798586 T>C and g.155798718 C>T of *PLPP3* promoter might be candidate molecular breeding markers for porcine follicular development. These works may provide valuable information for investigating the mechanisms of ovarian aging in mammals.

## Figures and Tables

**Table 1:** The genotype frequency and allele frequency of SNPs in the promoter of *PLPP3*.

Locus	Genotype	Genotype Frequency (Sample Size)	Allele Gene	Allele Frequency (Sample Size)
155798586T>C	TT	0.8169 (116)	T	0.9085 (258)
	CT	0.1831 (26)	C	0.09155 (26)
155798718C>T	CC	0.4859 (69)	C	0.7394 (210)
	CT	0.507 (72)	T	0.2606 (74)
	TT	0.007042 (1)		
155799380A>T	AA	0.7119 (42)	A	0.8559 (101)
	TA	0.2881 (17)	T	0.1441 (17)
155799135G>C	CC	0.661 (39)	C	0.8305 (98)
	CG	0.339 (20)	G	0.1695 (20)

**Table 2:** Correlation between different genotype of SNPs and the age at puberty.

Locus	Genotype	Sample Size	Average Age (d) ± Standard Deviation	*p*-Value
155798586T>C	TT	116	135.2 ± 10.994 ^a^	0.0002468
	CT	26	126.7 ± 7.499 ^b^	
155798718C>T	CC	69	138.7 ± 9.519 ^a^	0.0002468
	CT	72	129.0 ± 10.058 ^b^	
	TT	1	118.0 ± NA ^b^	
155799380A>T	AA	42	139.4 ± 9.069 ^a^	0.05365
	TA	17	134.2 ± 9.418 ^a^	
155799135G>C	CC	39	139.1 ± 9.189 ^a^	0.1742
	CG	20	135.6 ± 9.583 ^a^	

Note: *p* < 0.05 indicated a significant difference in the association between the SNP and age at puberty. All of the values were expressed as the least squares mean ± standard deviation. The “a” and “b” represented intra group differences.

## Data Availability

The RNA-seq data in this study have been deposited into the Sequence Read Archive database with the accession number PRJNA1143886. Any other data underlying this study will be provided by the corresponding authors upon reasonable request.

## Supplementary Materials

The following supporting information can be downloaded at: https://www.mdpi.com/article/10.3390/cells13171421/s1, Table S1. Primers for qRT-PCR; Table S2. Antibodies for immunofluorescence; Supplementary Table S3. Antibodies for Western blotting.

## Abbreviations

bp, base pair; DNA, deoxyribonucleic acid; GFP, green fluorescent protein; GCs, granulosa cells; HE, hematoxylin–eosin staining; MDA, malondialdehyde; mRFP, monomer red fluorescent protein; PBS, phosphate buffered saline; *PLPP3*, phospholipid phosphatase 3, qRT-PCR, real-time quantitative PCR; ROS, reactive oxygen species; RNA, ribose nucleic acid; siRNA, small interfering RNA; SA-β-gal, senescence associated β-Galactosidase; SNP, single nucleotide polymorphism; WB, Western blotting.
