# Supplementary material for: Phospholipid Phosphatase 3 (PLPP3) Induces Oxidative Stress to Accelerate Ovarian Aging in Pigs
Source: Cells. 2024 Aug 25;13(17):1421. doi: 10.3390/cells13171421 (PMC11394089; doi:10.3390/cells13171421)
Supplement: Supplementary file 1 [file cells-13-01421-s001.zip › cells-3090894-supplementary.pdf]

Supplementary Table S1. Primers for qRT-PCR.

Gene	Primer sequence (5' to 3')	Gene	Primer sequence (5' to 3')
PLPP3	F: CCTGCTGCATAGTTTTCTT<br>R: TGTTGTGGTGATTGTTCCT	*SIRT1*	F: ACCACCCACACCTCTTAAT<br>R: GACTCTCCATCGGTTCTTT
SOD1	F: GATTCTGTGATCGCCCTCT<br>R: CAGCATTTCCCGTCTTTGT	*SIRT6*	F: TGGACAATGGAGGAGCGAG<br>R: GACCAGGAAGCGGAGGAGG
SOD2	F: GGCTTTGGGGGTCCTGGGT<br>R: GCGTGGTGCTTGCTGTGGT	*p16*	F: GCGCCGTCTCTTGATTACTG<br>R: CTGGCTCCTCACTAGCAACA
CAT	F: CCAGCCAGTGACCAGATGAAG<br>R: ACACCTTCGCCTTCGAGAAT	*p21*	F: ACGTCTCAGGAGGACCATGT<br>R: AGAAGATCAGCCGGCGTTTG
CYP1B1	F: TTTCTCTTCATCTCCATCC<br>R: GGTCATTTTCCTCTGCTTG	*SLC7A11*	F: AATGGTGGTGTGTTTGCTGTCTC<br>R: GAGGAGTGTGTTTGCGGATGTG
p62	F: CGATGGCGATGTCGTATGT<br>R: TGCTGTGCTCCTTGTGAAT	*GPX4*	F: TTCCTCATTGATAAGAACGGCTGTG<br>R: GGTAGCACGGCAGGTCCTTC
ATG7	F: GGGCTGGCATCGCTTTGTA<br>R: GCAGGGTGGGCAGGTAGAA	*FTH1*	F: ATGACTGGGAGAATGGGCTGAC<br>R: ATCCAGGTAATGCGTCTCAATGAAG
ATG5	F: GCAAGTCCAGTCACTCTCG<br>R: ATTCACGCTCTCCGCTCAG	*NOX1*	F: CAGCATTCGCCAGTTCCAAGG<br>R: TCCAACCAACATAGCCACTTCATAC
BECN1	F: GGCAAGATTGAAGACACAG<br>R: AGGATACCCAAGCAAGACC	*FSP1*	F: TGGGGAAAAGGACGGATGAAGC<br>R: GCAGGACAGGAAGACGCAGTAC
LC3B	F: GAGCAGCATCCTACCAAAA<br>R: TCGTACACCTCACAAATCG	*GAPDH*	F: GGTCGGAGTGAACGGATTT<br>R: CCATTTGATGTTGGCGGGA

Supplementary Table S2. Antibodies for immunofluorescence.

Antigen	Host	Manufacture	Cat. number	dilution
PLPP3	Rabbit	Bioss	bs-4127R	1:300
SOD1	Rabbit	Affinity	AF5198	1:200
p62	Rabbit	Abcam	ab109012	1ug/mL

SIRT1	Rabbit	Proteintech	AB_10646436	1:300

Supplementary Table S3. Antibodies for western blotting.

Antigen	Host	Manufacture	Cat. number	dilution
PLPP3	Rabbit	Bioss	bs-4127R	1:300
SOD1	Rabbit	Affinity	AF5198	1:1000
CAT	Rabbit	Affinity	AB_2841044	1:1500
ATG7	Rabbit	Abcam	ab133528	1:20000
p62	Rabbit	Abcam	ab109012	1:20000
SIRT1	Rabbit	Proteintech	AB_10646436	1:3000
SIRT6	Rabbit	Proteintech	AB_2188915	1:1800
p21	Rabbit	Immunoway	YT3497	1:1000
LC3B	Rabbit	affinity	AF4650	1:1000
S100A4(FSP1)	Rabbit	Abmart	T55799	1:500
FTH1	Rabbit	Abmart	T55648	1:1000
SLC7A11	Rabbit	Abmart	T57046	1:1000
anti-Rabbit IgG	Rabbit	Abcam	ab205718	1:10000
α-Tubulin	Rabbit	Abcam	ab7291	1:10000
